# Texture Evolution during Recrystallization and Grain Growth in Non-Oriented Electrical Steel Produced by Compact Strip Production Process

**DOI:** 10.3390/ma15010197

**Published:** 2021-12-28

**Authors:** Jun-Qiang Cong, Fei-Hu Guo, Jia-Long Qiao, Sheng-Tao Qiu, Hai-Jun Wang

**Affiliations:** 1 School of Metallurgy and Resources, Anhui University of Technology, Maanshan 243002, China; mingh_cong@163.com (J.-Q.C.); qiustchina@126.com (S.-T.Q.); whjchina@ahut.edu.cn (H.-J.W.); 2 National Engineering Research Center of Continuous Casting Technology, China Iron & Steel Research Institute Group, Beijing 100081, China; guofeihu2020@163.com; 3 School of Metallurgy, Northeastern University, Shenyang 110819, China

**Keywords:** Compact Strip Production, non-oriented electrical steel, texture, recrystallization, grain growth, α*-fiber texture

## Abstract

Evolution of texture and α*-fiber texture formation mechanism of Fe-0.65%Si non-oriented electrical steel produced by Compact Strip Production (CSP) process during all the thermo-mechanical processing steps were investigated using electron backscatter diffraction (EBSD) and X-ray diffraction (XRD) techniques. Columnar crystal structure of cast slab is fine and well-developed. Textures of the hot-rolled band are quite different in the thickness direction. During annealing of cold-rolled sheet, γ-fiber texture grains would nucleate and grow preferentially, and α*-fiber texture grains mainly nucleate and grow in the shear zone of α-fiber texture of cold-rolled sheet. During the recrystallization process, γ-fiber texture gradually concentrated to {111}<112>, and γ and α*-fiber texture increased significantly. {111}<112> texture priority nucleation at the initial stage of recrystallization. Due to the advantages of nucleation position and quantity, the content of α*-fiber texture is greater than {111}<112> texture in the mid-recrystallization. During grain growth process, {111}<112> oriented grains would grow selectively by virtue of higher mobility, sizes and quantity advantages than that of {411}<148 > and {100}<120>, resulting in the gradual increase of γ-fiber texture and the decline of α *-fiber texture.

## 1. Introduction

Magnetic properties of non-oriented electrical steel are mainly influenced by chemical composition, microstructure and crystallographic texture [1]. Microstructure and crystallographic texture would determine domain wall pinning and motion, and these would fundamentally change several times during the thermo-mechanical processes. Due to domains being spontaneously magnetized to saturation in <100>, λ-fiber (<100>//ND) texture, which has two [001] directions, is the advantageous texture for magnetization. Inversely, γ-fiber (<111>//ND) texture, which has no [001] direction, becomes the adverse texture for magnetization. Thus, a proper control of microstructure and crystallographic texture is essential for improving magnetic properties in non-oriented electrical steels [2,3].

Numerous investigations [4,5,6,7,8,9,10,11,12] have already been conducted trying to optimize the processing parameters to eliminate the magnetically unfavorable texture while promoting the desired texture in non-oriented electrical steels. Increasing the shear band structure of the cold-rolled sheet is an effective way to promote <001>//ND texture of non-oriented electrical steel in the recrystallization process [13,14,15,16,17]. The Taylor model is also used to theoretically simulate the initial grain deformation process [18,19]. Meanwhile, lots of research has been done into the nucleation and growth of Cube, Goss and γ-fiber textures [20,21,22,23,24,25] and the formation of α*-fiber texture ({h 1 1}<1/h 1 2>) is mainly related to the deformation of {100}-oriented grains [26,27,28]. However, since the relationship between grain boundary energy, grain boundary migration speed and texture orientation have not been qualitatively concluded, the texture change law of the grain growth process needs to be further studied [2,3,29,30,31,32,33,34].

Simultaneously, the magnetic induction of the CSP process non-oriented electrical steel is about 0.02T higher than that of the conventional process [35], which provides a new technical approach for developing high-quality non-oriented electrical steel [35,36,37,38,39,40]. Consequently, evolution of microstructure and texture in recrystallization and grain growth process, and the formation mechanism of α*-fiber texture of the CSP process non-oriented electrical steel, have been studied in detail, with a view to provide a theoretical basis for further improving magnetic properties of CSP process non-oriented electrical steel.

## 2. Materials and Methods

The main chemical composition of CSP process Fe-0.65%Si non-oriented electrical steel is given in Table 1. The continuous casting billets with 70 mm in thickness were homogenized at 1100~1120 °C for 50 min, and were then hot rolled to 2.5 mm and coiled at 700~720 °C. Afterwards, hot-rolled bands were plastically deformed to 0.5 mm by tandem cold rolling. Finally, the cold-rolled sheets were annealed in 30%H_2_ + 70%N_2_ atmosphere with different temperatures in 3 min.

The microstructure was optically observed on the longitudinal section as defined by the RD and the ND using ZEISS-Axio Scope A1 optical microscope. Deformation and recrystallization macro-textures were measured at surface, quarter and center thickness layers using PANalytical EMPYREAN SERIES 2 X-ray diffraction with a radiation source of Co Ka. Micro-textures of the samples were analyzed by ZEISS SUPRA 55VP field emission scanning electron microscope equipped with EDAX OIM electron backscatter diffraction (EBSD) system. To define a particular texture component, a deviation angle of 15° was applied and the area fraction of various textures were calculated using OIM Analysis 6.1 software. In order to obtain more reliable data, a single sample randomly selected 2~4 areas for measurement, and the scanning step was selected to be less than 1/10 of the average grain size.

## 3. Results and Discussions

### 3.1. Evolution of Microstructure and Texture

**Figure 1:**
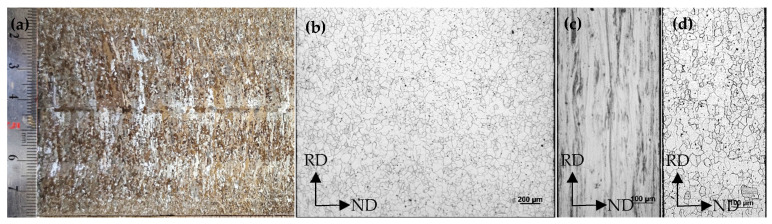
Microstructure morphology of CSP process Fe-0.65%Si non-oriented electrical steel billet (**a**), hot rolled band (**b**), cold rolled sheet (**c**) and annealed sheet (**d**).

Microstructure morphology of cast billet cross-section solidification, hot-rolled band, cold-rolled sheet and annealed sheet of CSP process Fe-0.65%Si non-oriented electrical steel are shown in Figure 1.

There is a certain degree of center segregation in the slab, and the inner and outer arc columnar crystals of the slab are fine and well developed, reaching the center. Microstructure of the hot-rolled band after coiling at 700~720 °C is ferrite, with an average grain size of 35.9μm. Cold-rolled sheet is a fibrous microstructure with a width of 10~55μm, and there is a large number of shear band structures with an angle of 20~30° to the rolling direction. After annealing at 820 °C for 2 min, grain boundaries of the annealed sheet are clear and regular, and the average grain size is 27.9μm.

**Figure 2:**
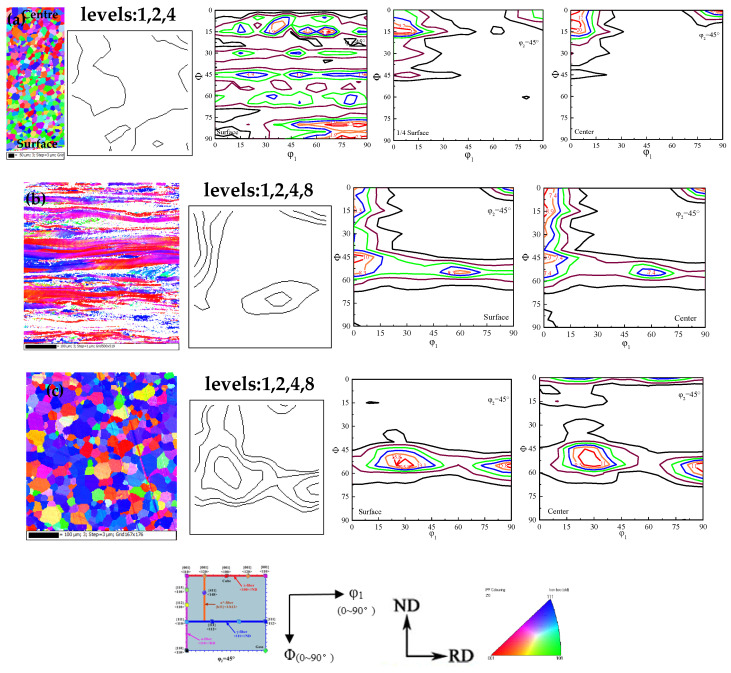
EBSD inverse pole figure (IPF) maps, micro-texture and macro-texture at different thickness layers in the φ_2_ = 45° of Fe-0.65%Si hot-rolled band (**a**), cold-rolled sheet (**b**) and annealed sheet (**c**).

**Figure 3:**
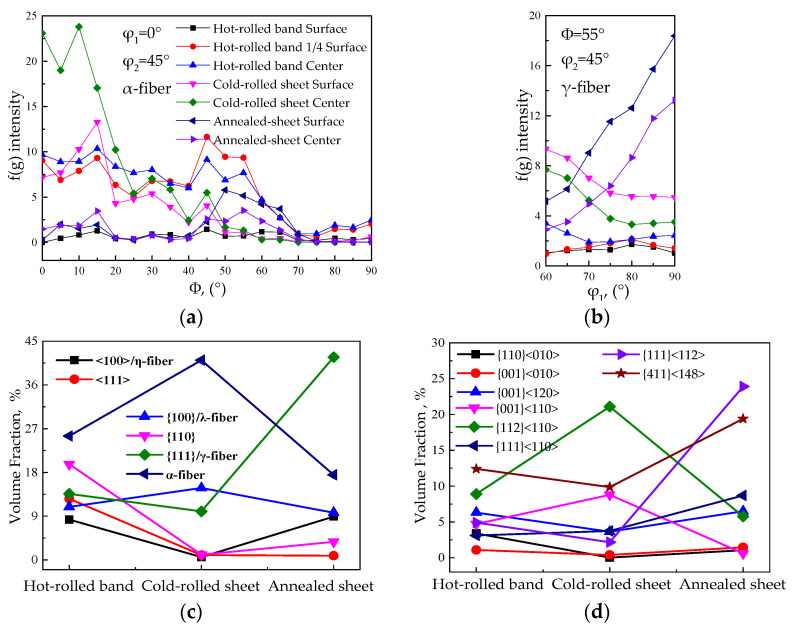
Orientation line distribution of α, γ textures and volume fraction distribution of textures of Fe-0.65%Si non-oriented electrical steel. (**a**) α textures, (**b**) γ textures, (**c**) and (**d**) volume fraction distribution of textures.

Figure 2 shows the EBSD inverse pole figure (IPF) maps, micro-texture and macro-texture ODF cross-sectional view of Fe-0.65%Si non-oriented electrical steel. Figure 3 shows distribution of α and γ orientation lines and content of specific texture components of a hot-rolled band, cold-rolled sheet and finished sheet in Fe-0.65%Si non-oriented electrical steel.

As shown in Figure 2 and Figure 3, textures in thickness of hot-rolled band are quite different, and a strong Goss texture is dispersed in the surface. Textures in 1/4-order surface layer are mainly α, γ-fiber texture and rotating-cube texture, and textures in the center layer mainly have a rotating-cube texture. Columnar crystals in continuous casting billet are well developed (as shown in Figure 1a), and columnar crystals would be compressed during the hot rolling process. Columnar crystals with the original orientation of <100> would rotate around the ND direction and deviate to the rotating cube orientation. Therefore, a certain intensity of rotating-cube texture would form during the hot rolling process. Finite element simulation study on crystal plastic deformation shows that the rotation behavior of columnar crystals with <100> orientation mainly depend on the accuracy of orientation. {100} orientation with 10° ≤ Δφ1 ≤ 20° and 0° ≤ ΔΦ ≤ 5° would be more inclined to rotate toward {001}<120>, and those with 10° ≤ Δφ1 ≤ 20° and 10° ≤ ΔΦ ≤ 15° would be more inclined to rotate toward {411}<148> during the rolling process [41]. Meanwhile, due to dynamic recrystallization during the hot rolling process, Goss (surface layer), γ and α-fiber textures would form [1,2,3], thus forming a texture distribution in the thickness direction of the hot-rolled band.

As the hot-rolled band was cold-rolled with a reduction rate of 80%, the cold-rolled sheet is mainly composed of {001}<110>, α and γ-fiber texture. Studies have shown that due to the presence of {110}<001> and a small amount of {001}<100>, oriented grains in the hot-rolled band would rotate during cold rolling; thus, *α* and *γ*-fiber texture would appear in a cold-rolled sheet [2,3,42]. In addition, during the cold rolling process, the initial grains tend to become {111}-oriented substructures with a high density of dislocations under the effect of a local slip near the grain boundaries [43]. As deformation increases, {111}<112> gradually turned to a more stable {111}<110> orientation, combining with the original inheritance of rotating-cube texture, forming a texture distribution of the cold-rolled sheet.

During the annealing process, recovery, recrystallization and grain growth occur and weaken the original deformation texture. {001}<120>, {411}<148> and *γ*-fiber texture are mainly composed textures of the annealed sheet. As energy storage of {111} and {112} grains are higher than that of {100}, {111} and {112} grains would nucleate and grown earlier and phagocytize {100}<011> component, resulting in a decrease of {100}<011>, forming a certain intensity of *γ*-fiber texture in the annealed sheet [2,3]. The recrystallized grains of α^*^-fiber texture ({h 1 1}<1/h 1 2>) were formed in the deformed tissue of *α*-fiber texture ({100}<011>~{112}<011>) in cold-rolled sheets during annealing [2,3]. Therefore, the grains of *γ*-fiber texture preferentially grow and the recrystallized, α^*^-fiber texture component would arise from {100}<011> to {112}<011> after the recrystallization of {111}<112> [2,3,41,44], and finally formed a typical annealed sheet texture.

### 3.2. Microstructure and Texture Evolution during Recrystallization

**Figure 4:**
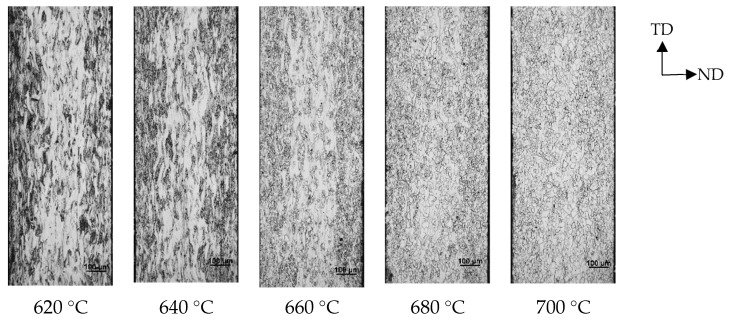
Microstructure of Fe-0.65%Si non-oriented electrical steel during recrystallization.

**Figure 5:**
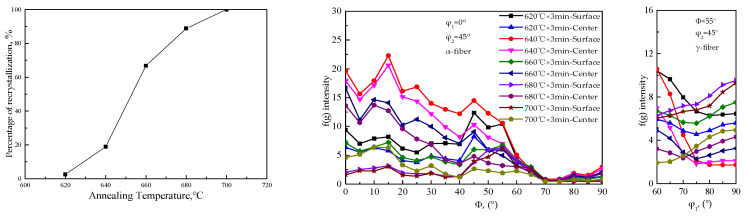
Relationship between recrystallization percentage and annealing temperature, orientation line distribution of α and γ fiber textures in Fe-0.65%Si non-oriented electrical steel during recrystallization.

Figure 4 and Figure 5 show the microstructure of the recrystallization process, variation of recrystallization percentage with annealing temperature, and the distribution of α and γ orientation lines, respectively. Cold-rolled sheet of Fe-0.65%Si non-oriented electrical steel would recrystallize at 620 °C and would be complete at 700 °C. As the recrystallization progresses, new grains nucleate along the shear band and gradually grow up. After complete recrystallization, the distribution of new grains at a specific angle to the rolling direction at the initial stage of recrystallization gradually disappears, and the microstructure is a regular ferrite microstructure.

**Figure 6:**
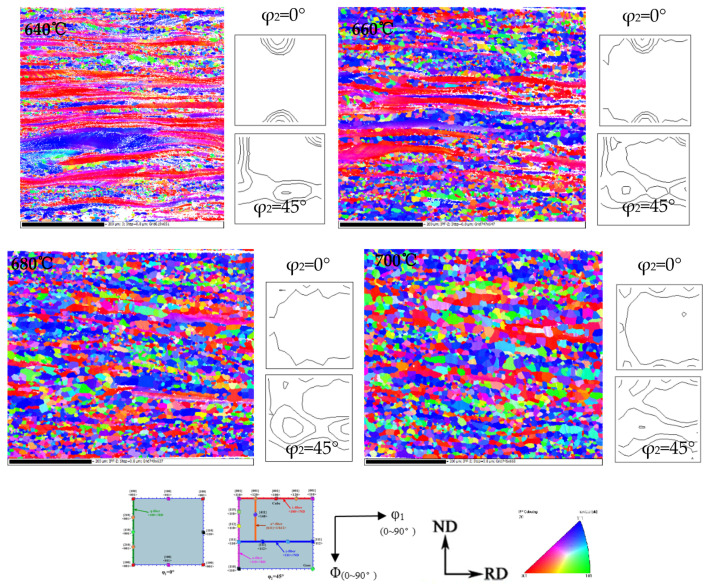
EBSD inverse pole figure (IPF) maps, φ_2_ = 0° and φ_2_ = 45° ODF of Fe-0.65%Si non-oriented electrical steel in the recrystallization process (levels: 1,2,4,8; deviation angle 15°).

**Figure 7:**
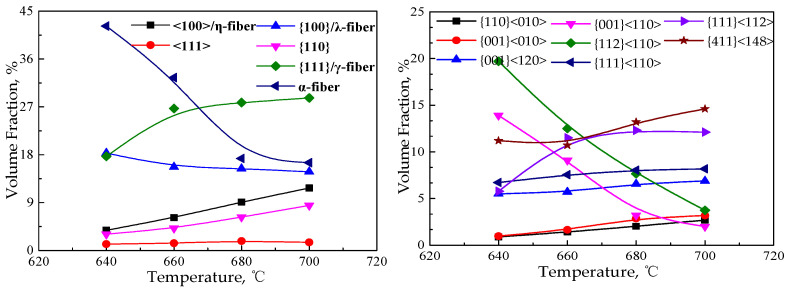
Texture in the annealed sheet of Fe-0.65%Si non-oriented electrical steel during recrystallization process (deviation angle 15°).

Cold-rolled sheet annealing at 620 °C has a lower proportion of recrystallization, and textures strength and type are basically the same as those of the cold-rolled sheet. With the progress of recrystallization, the strength of each texture shows a downward trend, and the texture type changes regularly. Figure 6 shows the EBSD inverse pole figure (IPF) maps and the thickness direction of the ODF cross-sectional view of Fe-0.65%Si non-oriented electrical steel in the recrystallization process. Micro-texture in the thickness direction of the recrystallization process corresponds to the macro-texture of different thicknesses (Figure 5). Figure 7 shows statistics of specific texture contents in the thickness direction of Fe-0.65%Si non-oriented electrical steel during the recrystallization process.

As the recrystallization progresses, γ-fiber texture is gradually concentrated to {111}<112>, and γ-fiber texture content gradually increases; α-fiber texture content decrease significantly, mainly reflect in content of {112}<110> and {111 }<112>. The content of the rotating-cube texture is reduced greatly, while that of Goss texture and cubic texture increases, reaching 2.68% and 3.17% after complete recrystallization, respectively. The content of α*-fiber shows an increasing trend, and {411}<148> and {100}<120> reach 14.6% and 6.88% as recrystallization was completed at 700°, respectively. The average grain size of the annealed sheet at 700 °C was 10.6 μm and that of {411}<148> and {111}<112> orientation grains were 13.1μm and 12.3μm, respectively.

**Figure 8:**
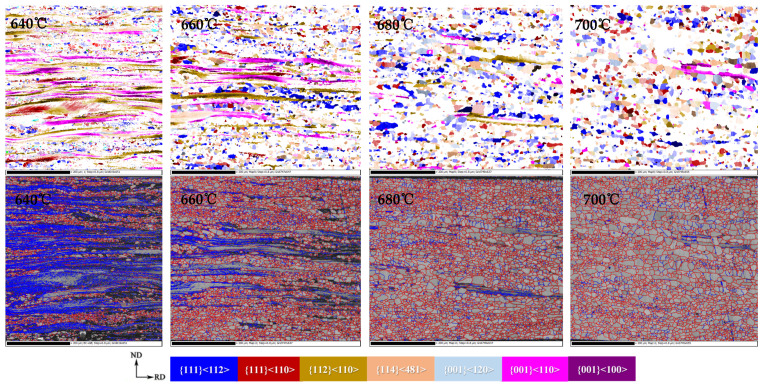
Textures and grain boundary distribution map of Fe-0.65%Si non-oriented electrical steel during recrystallization process.

Figure 8 shows the texture composition and grain boundary distribution of the CSP process non-oriented electrical steel during recrystallization. At the initial stage of recrystallization (640 °C), the recrystallized grains of α and γ-fiber texture first nucleate in the shear zone. As the energy storage of deformed grains in the {111}<112> orientation is higher than that in {111}<110> and {112}<110>, γ-fiber texture would nucleate in the shear band before α-fiber texture. Cubic-oriented and Goss-oriented grains also nucleate in the shear zone first, but the nucleation is not uniform and is relatively scattered. The main nucleation is in {001}<110>, { 112}<110>, {111}<110> and {111}<112> orientation-deformed grains. As {100}<011> has high content but low deformation energy, part of the {100}<011> texture would retain by in-situ recrystallization during annealing. Because of nucleation sites advantage of α*-fiber texture recrystallized grains, they would nucleate and grow mainly in the deformed tissue of α-fiber texture ({100}<011>~{112}< 011>).

Currently recognized texture formation theories are divided into directional nucleation theory [45], directional growth theory and directional nucleation-directional growth theory [46]. Analyzing the formation and transformation of the texture during the recrystallization process, formation and transformation of the texture are different at the initial stage of recrystallization. The types of recrystallized textures nucleate in the deformed grains of different orientations and the distribution of recrystallized grains is also uneven.

It shows that the type, percentage, number and distribution of grain nucleation texture are different on deformed grains with different orientations. With the progress of recrystallization, the changing trend of different textures are different, and are mainly manifested as the gradual increase of γ-fiber texture content, the obvious decrease of α-fiber texture, the slight increase of Goss and cubic texture content, and the rotating-cube texture greatly reduced and α* fiber texture content shows an increasing trend. Therefore, in the process of recrystallization, it is more appropriate to explain the formation mechanism of the recrystallized texture by the directional nucleation theory.

### 3.3. Microstructure and Texture Evolution during Grain Growth

**Figure 9:**
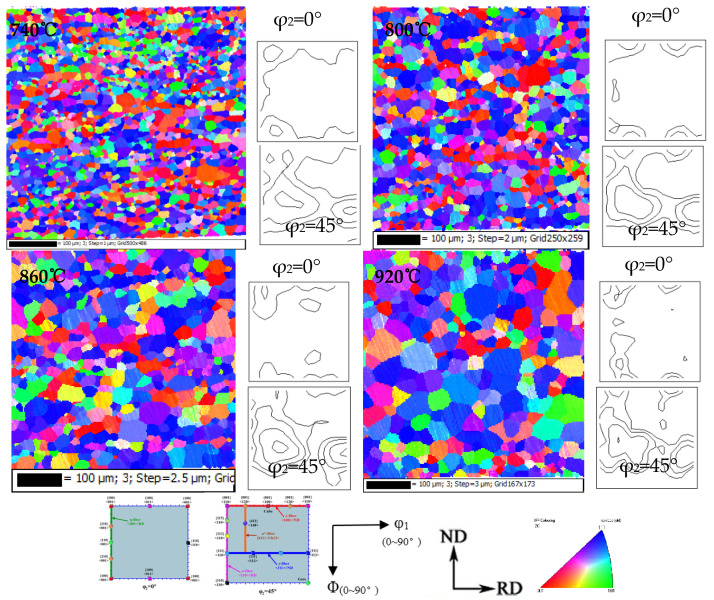
EBSD inverse pole figure (IPF) maps, φ_2_ = 0° and φ_2_ = 45° ODF sections of Fe-0.65%Si non-oriented electrical steel annealed sheet during grain growth (levels: 1,2,4,8; deviation angle 15°).

EBSD inverse pole figure (IPF) maps of each Fe-0.65%Si non-oriented electrical steel annealing sheet in the thickness direction during the grain growth process, and the ODF cross-sectional view of φ_2_ = 0° and φ_2_ = 45° are shown in Figure 9. With the increase of annealing temperature, the grain size increases obviously, and textures type in the thickness direction are basically unchanged, but texture content of γ-fiber increases significantly.

**Figure 10:**
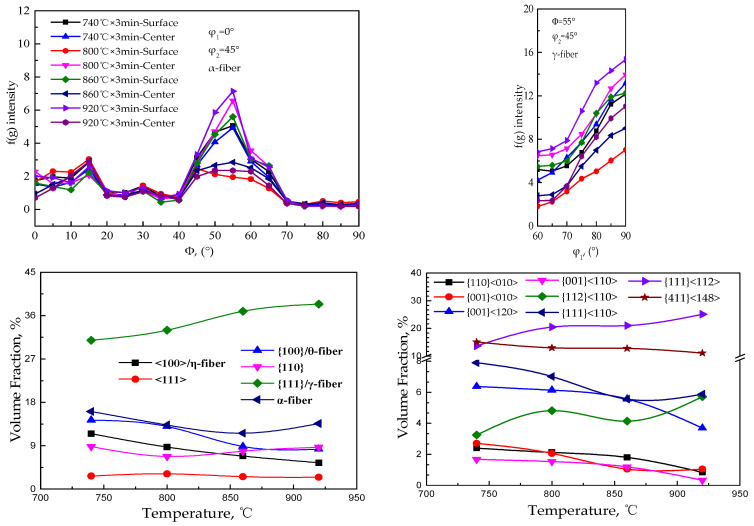
Orientation line distribution of α, γ fiber textures and distribution of texture content in Fe-0.65%Si non-oriented electrical steel annealed sheet during grain growth.

The orientation line distribution of α, γ-fiber texture and content distribution of specific texture in grain growth process of Fe-0.65%Si non-oriented electrical steel annealed sheet are shown in Figure 10. The grain growth process is usually accompanied by the migration of grain boundaries and a changing of texture.

**Figure 11:**
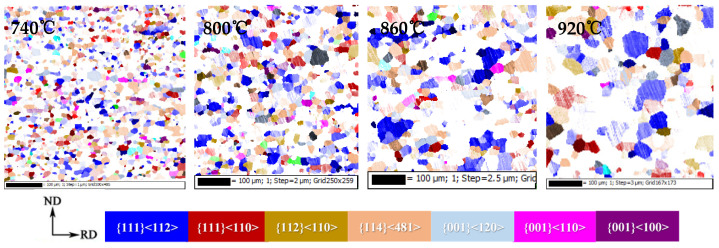
Texture component diagram during grain growth of Fe-0.65%Si non-oriented electrical steel annealed sheet.

With the growth of crystal grains, the main change in texture is reflected in the intensity change of specific texture, and is mainly concentrated on γ and α*-fiber textures, among which γ-fiber texture is mainly {111}<112>, and α*-fiber texture is mainly concentrated on {411}<148> and {001}<120>. With the increase in the annealing temperature, the texture content of <100>/η-fiber and {001}/λ-fiber decreases, γ-fiber increases, and α*- fiber decreases. Figure 11 shows the distribution diagram of specific texture components in the annealed sheets during the grain growth of the CSP process Fe-0.65%Si non-oriented electrical steel.

According to the statistics of the grain boundary angle of the grains along the thickness direction after annealing at 740–920 °C, it was found that the large-angle grain boundary accounts for 73%–81%, and the small-angle grain boundary does not appear to be concentrated in a specific orientation grain trend. It is generally considered that the large-angle grain boundary with an orientation difference of more than 15° and less than 45° with the adjacent grains is a high-mobility grain boundary, and the recrystallization stages of the grains with a large-angle grain boundary are basically completed.

Therefore, it is speculated that the grain growth rate of each orientation during the grain growth process is similar, and the percentage of each grain remains stable with the increase of temperature. However, since there are more original recrystallized grains in {111}<112> and {411}<148> orientations, average size of {111}<112> and {411}<148> oriented grains are significantly larger, which reflects the difference in the growth rate of grains with a specific orientation during the grain growth stage.

### 3.4. α*-Fiber Texture Formation Mechanism

**Figure 12:**
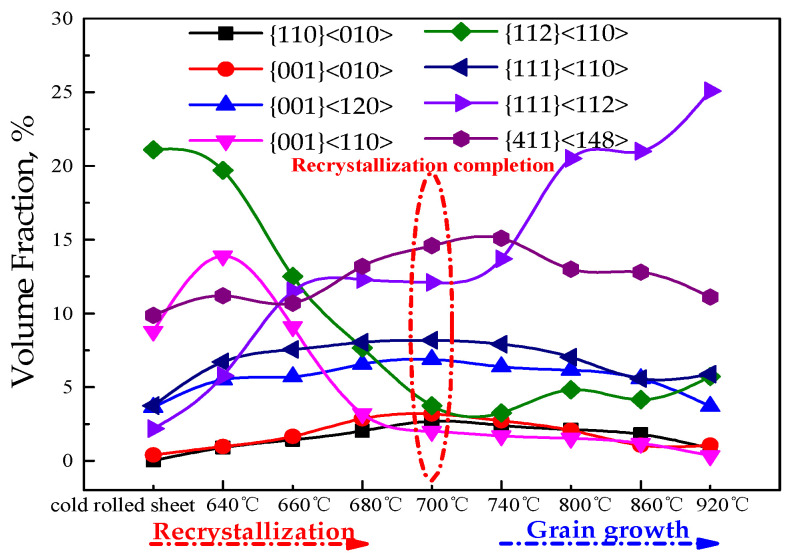
Variation trend of main texture components content in recrystallization and grain growth process of CSP process Fe-0.65%Si non-oriented electrical steel annealed sheets.

Increases in α*-fiber texture ({h 1 1}<1/h 1 2>) is usually accompanied by a decrease in γ-fiber texture, which is beneficial to improve the magnetic properties [1,2,3]. Statistical main texture content changes in the recrystallization and grain growth process of the CSP process Fe-0.65%Si non-oriented electrical steel annealed sheets are shown in Figure 12. During the recrystallization process, the occupancy rate of γ and α*-fiber texture is gradually increased, Goss and cubic textures showed an increasing trend, rotating-cube texture and α-fiber texture decreased greatly. In the process of grain growth, γ-fiber texture gradually increases, and α*-fiber texture shows a downward trend. Therefore, {111}<112>, {411}<148> and {100}<120> texture are selected for comparative analysis to study the formation mechanism of α*-fiber texture.

Regarding the nucleation characteristics of {111}<112> oriented grains [1,2,3], the early stage of recrystallization preferentially nucleates near the grain boundaries and inside {111}<uvw> deformed grains, {111}<112> deformed grains with high stored energy would preferentially nucleate. The {411}<148> and {100}<120> orientation grains mainly nucleate near the {100}<011>~{112}<110> textures with a higher content. Therefore, {411}<148> and {100}<120> orientation grains have more nucleation positions in the subsequent recrystallization process, and have an advantage in the number of nucleation.

**Figure 13:**
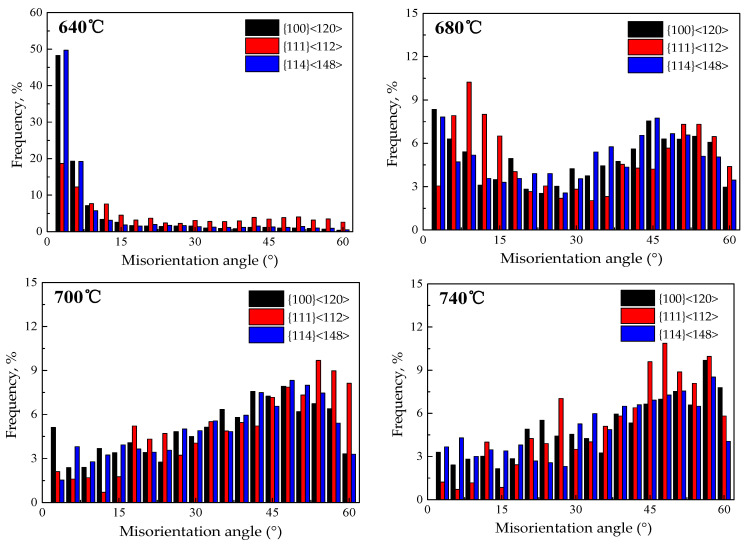
Distribution of orientation difference between {100}<120>, {111}<112> and {411}<148> textures and their adjacent grains of CSP process Fe-0.65%Si non-oriented electrical steel annealed sheets.

**Figure 14:**
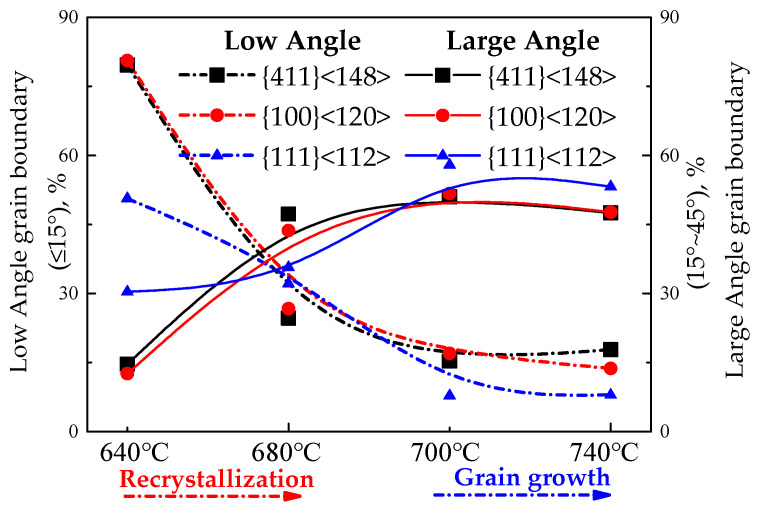
Angle distribution of grain boundaries between specific orientation grains and adjacent grains of CSP process Fe-0.65%Si non-oriented electrical steel annealed sheets, %.

Statistics concerning the angle distribution between {111}<112>, {411}<148>, {100}<120> textures and their adjacent grain orientation in recrystallization and grain growth processes of Fe-0.65%Si non-oriented electrical steel annealed sheets are shown in Figure 13 and Figure 14.

Annealing at 640 °C, {111}<112> oriented grains with low-angle grain boundaries between adjacent grains (orientation difference with adjacent grains less than 15°) account for 50.64%, the ratio of low-angle grain boundaries between oriented grains and adjacent grains of {411}<148> and {100}<120> are 79.66% and 80.6%, respectively. {411}<148> and {100}<120> oriented grains account for 14.65% and 12.6% of high mobility grain boundaries (the difference in orientation with adjacent grains is greater than 15° and less than 45° grain boundaries), respectively; {111}<112> oriented grains accounted for 30.37% of high mobility grain boundaries. Therefore, in the early stage of recrystallization, {111}<112> texture precedes {411}<148> and {100}<120> textures to recrystallize. With the progress of recrystallization, the ratio of {411}<148> and {100}<120> recrystallization texture is greater than {111}<112> orientation recrystallization texture due to the advantages of nucleation location and quantity.

The average grain size has increased to 11.4 μm in the 740 °C annealing sheet, and that of {411}<148> and {100}<120> oriented grains are 12.9 and 12.1μm, respectively, which are smaller than {111}<112> oriented grains (the average grain size is 14.5μm). After annealing at 740 °C, the proportion of low-angle grain boundaries of {111}<112> is 7.95%, and low-angle grain boundaries of {411}<148> and {100}<120> are 17.8% and 13.71%, respectively. Compared with the recrystallization process, the low-angle grain boundaries have decreased, but the low-angle grain boundaries of {411}<148> and {100}<120> are higher than that of {111}<112>. The proportions of high mobility grain boundaries of {411}<148>, {100}<120> and {111}<112> are 47.5%, 47.67% and 53.2%, respectively.

The texture evolution of {411}<148> and {100}<120> regarding the grain growth process could be explained by the influence of driving force and mobility during normal grain growth. Studies [47] on the process of grain growth showed that as far as the driving force is concerned, the certain orientation of grain size larger than the critical size of the matrix grain would grow due to a positive growth rate. In addition, with respect to adjacent crystal grains, low-angle grain boundaries are considered to have a lower mobility, and the more low-angle grain boundaries are, the less likely they are to migrate. During the growth of recrystallized grains, the total grain boundary area and grain boundary energy per unit volume decreased with the increase of grain size. Therefore, the reduction of grain boundary energy could be the driving force for grain growth. There would always be a difference in the size of each crystal grain in a ferrite, and the larger the difference in the sizes between adjacent crystal grains is, the easier it is for the large-size grains to swallow the small-size grains and to grow.

{411}<148>, {100}<120> and {111}<112> orientation grains would appear to grow rapidly after completely recrystallized, and their average grain sizes are larger. Therefore, it occupies a higher size advantage, and it is easier to swallow other small crystal grains during the growth process. From the perspective of misorientation, low-angle grain boundaries between {411}<148>, {100}<120>, {111}<112> and adjacent grains are gradually decreasing, which means that they are more likely to occur in migration, but because {111}<112> has fewer low-angle grain boundaries and a higher mobility, selective growth appears due to the advantages of high mobility and size.

In summary, {111}<112> texture in the early stage of recrystallization recrystallizes before {411}<148> and {100}<120>, and the content of α*-fiber is greater than {111}<112> orientation recrystallization texture in the middle of recrystallization. During the grain growth process, {111}<112> grains would grow selectively by virtue of higher mobility, sizes and quantity advantages than that of {411}<148> and {100}<120>.

## 4. Conclusions

Evolution of textures and α*-fiber texture formation mechanism of Fe-0.65%Si non-oriented electrical steel produced by CSP process during recrystallization and grain growth process were investigated. The main conclusions are:

(1) Columnar crystals in continuous casting billet are small and well developed, and reach the center. The texture of the hot-rolled band varies greatly in the thickness direction. Textures of the cold-rolled sheet are mainly γ- and α-fiber texture. γ-fiber texture grains preferentially nucleate and grow, and α*-fiber texture recrystallized grains would nucleate and grow in the deformed structure of α-fiber texture in the annealed sheet.

(2) With the progress of recrystallization, γ-fiber texture gradually concentrates towards {111}<112>, and γ and α*-fiber texture increases greatly. {111}<112> content gradually increases and that of {411}<148> and {001}<120> show a downward trend in the process of grain growth.

(3) {111}<112> would recrystallize before {411}<148> and {100}<120> in the early stage of recrystallization, and the content of α*-fiber texture is higher than {111}<112> in the middle stage of recrystallization. During grain growth, {111}<112> grains would grow selectively by virtue of higher mobility, sizes and quantity advantages than that of {411}<148> and {100}<120>.

## Figures and Tables

**Table 1:** The composition of Fe-0.65%Si non-oriented electrical steel in CSP process (wt %).

Element	C	Si	Mn	P	S	Al	N	Cu	Ti
Content	0.0028	0.65	0.25	0.075	0.0038	0.30	0.0035	0.031	0.0029
Composition deviation	±0.0002	±0.01	±0.01	±0.005	±0.0002	±0.01	±0.0002	±0.005	±0.0002

## Data Availability

Not applicable.
